# Ultrasonic reflection coefficient and surface roughness index of OA articular cartilage: relation to pathological assessment

**DOI:** 10.1186/1471-2474-13-34

**Published:** 2012-03-10

**Authors:** Hai-jun Niu, Qing Wang, Yue-xiang Wang, De-yu Li, Yu-bo Fan, Wu-fan Chen

**Affiliations:** 1 Key Laboratory of the Ministry of Education for Biomechanics and Mechanobiology, School of Biological Science and Medical Engineering, Beihang University, Beijing 100191, China; 2 School of Biomedical Engineering, Southern Medical University, Guangzhou 510515, China; 3 Department of Ultrasound, People Liberation Army General Hospital, Beijing 100853, China

**Keywords:** Osteoarthritis, High-frequency ultrasound, Ultrasound roughness index, Ultrasound reflection coefficient

## Abstract

**Background:**

Early diagnosis of Osteoarthritis (OA) is essential for preventing further cartilage destruction and decreasing severe complications. The aims of this study are to explore the relationship between OA pathological grades and quantitative acoustic parameters and to provide more objective criteria for ultrasonic microscopic evaluation of the OA cartilage.

**Methods:**

Articular cartilage samples were prepared from rabbit knees and scanned using ultrasound biomicroscopy (UBM). Three quantitative parameters, including the roughness index of the cartilage surface (URI), the reflection coefficients from the cartilage surface (R) and from the cartilage-bone interface (R_bone_) were extracted. The osteoarthritis grades of these cartilage samples were qualitatively assessed by histology according to the grading standards of International Osteoarthritis Institute (OARSI). The relationship between these quantitative parameters and the osteoarthritis grades was explored.

**Results:**

The results showed that URI increased with the OA grade. URI of the normal cartilage samples was significantly lower than the one of the OA cartilage samples. There was no significant difference in URI between the grade 1 cartilage samples and the grade 2 cartilage samples. The reflection coefficient of the cartilage surface reduced significantly with the development of OA (p < 0.05), while the reflection coefficient of the cartilage-bone interface increased with the increase of grade.

**Conclusion:**

High frequency ultrasound measurements can reflect the changes in the surface roughness index and the ultrasound reflection coefficients of the cartilage samples with different OA grades. This study may provide useful information for the quantitative ultrasonic diagnosis of early OA.

## Background

Osteoarthritis (OA) is a chronic joint disease that occurs with the progressive degeneration of articular cartilage followed by secondary bone hyperplasia. Clinical symptoms of OA include joint pain, function loss and joint deformity [1,2]. Early diagnosis of OA is essential for the timely treatment and prevention against advanced complications [3].

Techniques such as arthroscopy, X-ray computed tomography (CT) and magnetic resonance imaging (MRI) have been used in studies on articular cartilage and OA assessment [4-10]. Arthroscopy is usually used to qualitatively observe the visual changes in the superficial layer of articular cartilage. However, the degenerative changes in the deeper layers of the cartilage tissue are revealed by using this method with difficulty. Due to the principle of X-ray imaging, CT is effective in revealing bone degeneration but insensitive to soft tissue without contrast agents. MRI, which is considered as the most promising diagnostic technique, can noninvasively detect the cartilage surface contour, tissue compositions, and collagen orientation. However, clinical MRI imaging remains expensive and its relative long time of imaging and low resolution is limited to cartilage fine structure.

As a cheap, easy-to -use testing method and experimental means, ultrasonic measurement technique has been paid much attention in the research for the early diagnosis of OA in recent years. Many researchers measured the deformation of the cartilage tissue during the compression or swelling of articular cartilage using high-frequency ultrasound and input the experimental data to the single-phase, biphasic, and triphasic models to investigate the altered mechanical properties of the degraded cartilage [11-17]. Other researchers applied high-frequency ultrasound to explore the relation between the acoustic parameters (such as velocity, attenuation, etc.) and the composition changes in the progress of the cartilage degradation [18-20]. Previous studies have shown the potential of high frequency ultrasound to detect the cartilage degeneration.

Clinically, the severity of OA is usually graded in accordance with the morphological changes of the cartilage surface (such as degree of roughness and fibrillation) and the histopathological assessment of the tissue sections [15,21]. This method could provide more accurate grading with the observation of the changes in the cartilage surface and the inside tissue. Therefore, histopathological score of OA has been accepted as a "gold standard" for assessment of cartilage lesions in OA and moreover for validation of other assessment methods such us arthroscopy, ultrasound and MRI.

Ultrasound biomicroscopy (UBM) is a high-resolution ultrasound imaging system that can be used to observe the changes of the internal fine structure of the soft tissue. Previous results showed that there was a significant correlation between the UBM image and the pathological image of arthritis cartilage with different grades [15]. Unfortunately, no quantitative indicators were given in that study. As mentioned above, the morphological changes on the cartilage surface and the composition changes of articular cartilage are important bases for clinical grading of arthritis. Meanwhile, the roughness index can describe the morphological changes of the cartilage surface and the alterations in acoustic characteristics follow the composition changes of articular cartilage. One of the most intuitive alterations in acoustic characteristics is the changes in the reflection coefficient. Therefore, this study employed the UBM technique and calculated the roughness index of cartilage surface and the reflection coefficient of the cartilage tissue. The aims of this study are to explore the relationship between OA pathological grades and quantitative acoustic parameters and to provide more objective criteria for ultrasonic microscopic evaluation of the OA cartilage.

## Methods

### Animal models

Eighteen normal adult New Zealand white female rabbits weighing 3.5 to 4.5 kg (mean, 4.1 ± 0.3 kg) were used in this study. Radiographs of both femorotibial joints were taken and evaluated by two orthopedists to exclude animals with joint pathology. Six rabbits were treated as control. To induce OA, surgical transection of the anterior cruciate ligament (ACL) in the left femorotibial joint was performed under general anesthesia in twelve rabbits. Routine skin incision closure was performed. Antibiotics (penicillin 20,000 IU) were injected intramuscularly twice a day preoperatively and for 2 days postoperatively in the operated animals. Following surgery, free movement was allowed in separate cages for the duration of the experimental period. Experiments on the rabbits were approved by our institutional animal care and use committee and performed under the guidelines of the National Institutes of Health for the care of laboratory animals.

At 2, 4, and 6 weeks after surgery, six animals were euthanized, including two control rabbits and four rabbits with ACL transection. Each of the left knees was dissected and sectioned with a band saw to obtain the articular cartilage samples with pathological characteristics. 72 cartilage specimens were collected, including 18 medial femoralcondyles, 18 lateral femoral condyles, 18 medial tibial plateaus, and 18 lateral tibial plateaus. All the specimens were wrapped in wet gauze soaked with saline buffer and stored at -20°C until the ultrasound examination.

### Ultrasound examination

UBM imaging was performed on these cartilage surfaces using a Vevo770 (VisualSonics, Toronto, ON, Canada) high-frequency UBM system. A transducer (RMV708) with a nominal central frequency of 55 MHz was used. This transducer has a focal length of 4.5 mm and an axial resolution of 30 μm.

**Figure 1:**
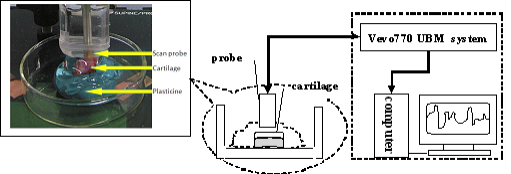
**Schematic of the ultrasound experimental set-up using Vevo 770 UBM system**. Samples were fixed at the bottom of the container by plasticine and the cartilage surface was covered with ultrasound gel.

Before ultrasound scan, each specimen was first assessed macroscopically by two radiologists [15]. Then the area on the cartilage surface with the most severe macroscopic lesion was selected to be scanned by ultrasound. The specimen was fixed using plasticine to ensure a horizontal cartilage surface of the ultrasound-scanned site (Figure 1). Gel was then put on the surface of the cartilage. A clamp that could be adjusted in the vertical and lateral directions was used to fix the transducer vertically to the cartilage surface. By adjusting the clamp, the transducer surface was immerged into gel and was placed approximately 4.5 mm above the cartilage surface with the focal zone of ultrasound beam located inside the cartilage layer to obtain the maximum echo amplitude. Two-dimensional UBM images were acquired with a 4 × 4 mm^2 ^field of view at 46 frames/s. The data of the ultrasound radiofrequency (RF) signals were recorded and stored for the calculation of the acoustic parameters of the cartilage tissue.

### Pathological grade

According to the grading standards of International Osteoarthritis Institute (OARSI), OA is graded as follows: grade 1 = uneven surface that can demonstrate superficial fibrillation; grade 2 = surface discontinuity that may be accompanied by cell proliferation, increased or decreased matrix staining in the mid zone; grade 3 = vertical fissures extending into the mid zone or erosion; grade 4 = denudation (the unmineralized hyaline cartilage is completely eroded); grade 5 = deformation. OA develops with three stages: early stage (grade 1-2), intermediate stage (grade 3) and advanced stage (grade 4-5) [15,21].

Cartilage samples used for pathological observation were fixed in 10% neutral buffered formalin, decalcified with 14% ethylenediamine tetra-acetic acid, dehydrated through graded alcohols, cleared with toluene, and embedded in paraffin. Careful attention was paid to make the histologically assessed site consistent with the ultrasound-scanned site. Six-micrometer sections of articular cartilage were cut from the proximal part to the ultrasound-scanned site. The sections were stained with toluidine blue. Then the sections were observed and graded by three board-certified pathologists blinded with the purpose of the whole study.

### Parameter extraction

In this study, three parameters are extracted to quantitatively describe the acoustic properties of articular cartilage, i.e. ultrasound roughness index (URI) of the cartilage surface, reflection coefficient (R) of the cartilage surface, reflection coefficient (R_bone_) of the cartilage-bone interface. URI is used to describe the microtopography of the cartilage surface. R and R_bone _are used to describe characteristics of cartilage tissue [11].

**Figure 2:**
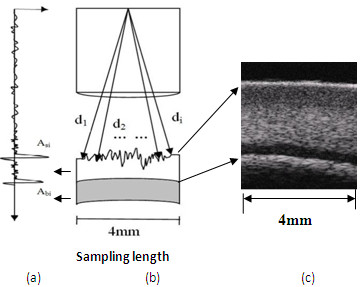
**Schematic of calculation of ultrasound roughness index (URI)**. (a) Ultrasound RF signal reflected from the articular cartilage tissue; (b) Schematic of the scanning scope of ultrasound probe; (c) An ultrasound microscopic image of cartilage tissue.

As shown in Figure 2, URI is obtained from the echo signals between the probe and the cartilage surface. It is calculated using equation 1.

(1) $$URI=\sqrt{\frac{1}{m}\;\;\overset{m}{\underset{i=1}{\sum }}{\left(di-\left\langle d\right\rangle \right)}^{2}}$$

where *m *is the number of the scan lines in the 4 mm sampling length. *d*i is the distance between the transducer and the solution- cartilage interface in the scan line *i*, and 〈*d*〉 is the average distance between the transducer and the surface.

R and R_bone _are defined by equation 2 and equation 3, respectively.

(2) $$R=\frac{1}{m}\overset{m}{\underset{i=1}{\sum }}\frac{A_{si}}{A_{iref}}$$

(3) $$R_{bone}=\frac{1}{m}\overset{m}{\underset{i=1}{\sum }}\frac{A_{bi}}{A_{iref}}$$

where *A_si_*and *A_bi _*are the peak-to-peak amplitude of the ultrasound RF signals reflected from the cartilage surface and the cartilage-bone interface, respectively, in the scan line *i*. *m *is the number of scan lines. *A_iref _*is the reference peak-to-peak amplitude of the echoes reflected from the solution-air interface with the same distance as *A_si_*. In the parameter calculation, the ultrasound speed in the solution is 1500 m/s, and the average ultrasound speed in the cartilage tissue equals 1600 m/s [11]. The analysis of ultrasound echo signals and the extraction of the ultrasound parameters were carried out by a custom-designed Matlab program (MATLAB, Version 2009, The Math-Works, USA).

### Statistical analysis

Statistical analyses were conducted with SPSS software (Version 17, SPSS Inc., USA). All values in the text are presented as mean ± standard deviation (SD). All specimens were evaluated to the normal, grade 1, grade 2, grade 3, grade 4, grade 5 groups. The analysis of variance in URI, R, R_bone _between grading groups were performed using One-way ANOVA and LSD post-hoc tests. P < 0.05 was taken as statistically significant.

## Results

This study evaluated 72 articular cartilage samples according to the OARSI grading system. As listed in Table 1, 29 samples were normal, while abnormality was found in 43 cartilage samples, including 14 grade 1 cartilage samples, 23 grade 2 cartilage samples, and 6 grade 3 cartilage samples. There was no grade 4 and grade 5 cartilage samples.

**Table 1:** OARSI grades of articular cartilage samples and ultrasound parameters

Ultrasound Parameters			
**OARSI grade**			

	**Uri(μm)**	**R(%)**	**R_bone_(%)**

Normal (N = 29)	10.36 ± 5.82	9.05 ± 1.78	19.314 ± 5.46

grade 1 (N = 14)	69.55 ± 27.43	5.22 ± 2.13	19.81 ± 7.33

grade 2 (N = 23)	76.86 ± 39.20	3.90 ± 2.07	21.87 ± 8.82

grade 3 (N = 6)	193.27 ± 75.06.	1.08 ± 0.85	30.33 ± 6.19

OARSI = Osteoarthritis Research Society International;

N is the number of the articular cartilage samples.

**Figure 3:**
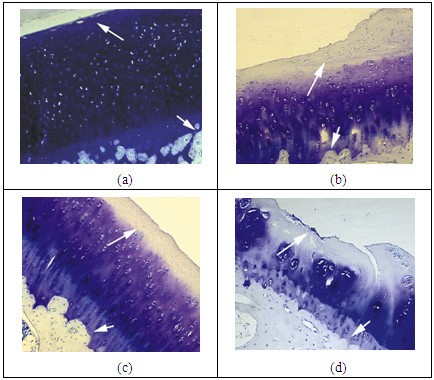
**The toluidine blue stained histological sections of (a) normal cartilage, (b) the grade 1 cartilage, (c) the grade 2 cartilage, and (d) the grade 3 cartilage**. Matrix staining was homogeneously colored by toluidine blue in (a). The reduction in staining was found in (b-d). The long arrows indicate the superficial zone of articular cartilage. The short arrows show the interface between the bone and the cartilage.

Figure 3 shows the pathological sections (toluidine blue staining) of normal cartilage and three cartilage samples with different OA grades. The pathological sections were taken from the same sites where were scanned by UBM. Figure 3a shows a sample of normal cartilage. The cartilage surface is flat and smooth. The full cartilage matrix is homogeneously stained by toluidine blue. The OA cartilage samples with grade1, 2 and 3 are respectively showed in Figure 3b-d. The surface of the grade 1 cartilage sample became uneven with a slight appearance of fibrillation and was slightly stained by toluidine blue. The toluidine blue staining of the surface of the grade 2 cartilage sample completely disappeared, and the cartilage surface was not flat. An obvious cartilage thinning was found in the OA cartilage with grade 3. The toluidine blue staining of the superficial and mid layers disappeared and the deep tissue was involved into the degradation.

**Figure 4:**
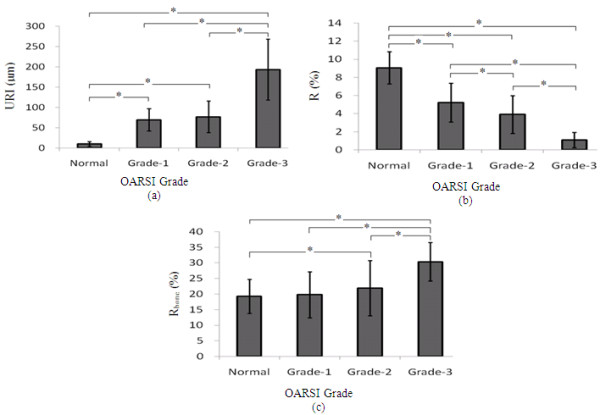
**The results of the statistical analysis of URI, R of the cartilage surface, and R_bone _of the cartilage-bone surface of the OA cartilage samples with different grades in comparison with normal cartilage samples**. * denotes significant difference at p < 0.05.

Table 1 lists the parameters extracted from the ultrasonic echo signals, including URI of the cartilage surface and the reflection coefficients of the cartilage surface and the cartilage-bone interface. It was found that URI of the cartilage surface increased with the OA grade (Table 1 Figure 4a). URI of the normal cartilage samples (10.36 ± 5.82 μm) was significantly lower than that of the OA cartilage samples (grade 1 cartilage: 69.55 ± 27.43 μm; grade 2 cartilage: 76.86 ± 39.20 μm; grade 3 cartilage: 193.27 ± 75.06 μm) (p < 0.05). Additionally, URI of the OA cartilage with grade 3 is significantly higher than that of the OA cartilage with grade 1 and 2 (p < 0.05), while there was no significant difference in URI between the grade 1 cartilage samples and the grade 2 cartilage samples.

Figure 4b shows the reflection coefficient of the cartilage surface of normal and OA cartilage samples, indicating that the reflection coefficient reduced significantly with the development of OA (p < 0.05). There were significant differences between normal and OA cartilage (p < 0.05), and significant intergroup differences among the samples with different grades (p < 0.05).

However, the reflection coefficient of cartilage-bone interfaces showed a gradual increase with the increase of grade (Figure 4c). R_bone _of the grade 1 cartilage samples is close to that of normal cartilage. There were no significant difference between the grade 1 cartilage and the grade 2 cartilage (p > 0.05). Both groups of the grade 2 cartilage samples and the grade 3 cartilage samples had a significant increase in R_bone _in comparison with normal cartilage (p < 0.05).

## Discussion

In this study, the reflection coefficients of the cartilage tissue and the roughness index of the cartilage surface were quantitatively described using the ultrasound microscopic imaging, and the relationship between the quantitative acoustic parameters and the severity of OA cartilage qualitatively assessed by histology was explored.

The results of our histological assessment indicate that the pathological symptoms in OA cartilage become more obvious and serious with the increase of OA grade. The cartilage surface became more uneven, the fibrillation of the superficial tissue more serious, and toluidine blue staining faded or totally disappeared from the surface to the deep layers as OA grade increased. The cartilage thickness decreased in the grade 3 cartilage samples. Previous studies reported consistent results that the proteoglycan content of articular cartilage changed first in the early OA, decreasing gradually from the surface to the deeper layers with the degeneration [22,23].

It has been well known that the earliest signs of OA include the loss of proteoglycans in the superficial layer and the disruption of the superficial collagen network, leading to fibrillation in the surface and softening in the superficial tissue [24-26]. High-frequency UBM has been demonstrated its useful application in detecting the early damage in the articular cartilage tissue due to the ability of ultrasound to penetrate into the tissue [15]. Responding to the changes in the surface roughness and the internal compositions of the OA cartilage, the ultrasound reflection and scattering in the tissue increased and thus the enhanced echoes reflected from the internal tissue were demonstrated in the UBM image [15]. The finding is consistent with the results of other studies [12,27]. These studies, however, did not provide any quantitative criteria for the ultrasonic diagnosis of OA cartilage.

In engineering, the roughness index is mainly used to describe the small valley and pitch conditions on the material surface, as known as micro-roughness. Recently, it has been used to describe the roughness of the cartilage surface in the evaluation of cartilage degeneration. Our results of the surface roughness calculation show that the roughness of the cartilage surface increased with the OA grade, indicating that the cartilage surface became rough and uneven with the development of OA. Similar results have been obtained in previous studies of cartilage degradation [11]. The statistical results in this study showed significant differences in URI between the grade 3 cartilage and the cartilage with grade 1 and grade 2 (p < 0.05), but an insignificant change between the grade 1 cartilage and the grade 2 cartilage (p > 0.05), showing that cartilage degradation progressively developed from quantitative change to qualitative change. It was found that the surfaces of the cartilage samples with grade 1 and 2 started to become rough with a significant increase in URI compared with normal cartilage, but no significant change between groups of grade 1 and grade 2. However, a sharp deterioration occurred in the grade 3 cartilage samples with a great increase in URI to 193.27 ± 75.06 μm. The results show that the surface roughness index URI could be used to distinguish early OA and mid OA, but has no ability to accurately distinguish the grades of early OA (i.e. grade 1 and grade 2).

Two possible reasons may explain the decrease in the reflection coefficient of the cartilage surface with the OA grade. First, the increase of surface roughness resulted in diffuse reflection and consequently decreased the amplitude of the echoes. Secondly, while OA occurred, the cartilage surface was softened. The compositions and structure of articular cartilage gradually changed from the surface to the deep layer. As more transmitted ultrasonic energies were absorbed, the reflection coefficient decreased. Contrary to the reflection coefficient of the surface, the reflection coefficient of the cartilage-bone interface increased with OA grades (Figure 4c). It was revealed that the bottom reflection coefficient of the grade 1 cartilage changed insignificantly compared with normal cartilage while that of OA cartilage with grade 2 and 3 increased significantly. The OA cartilage with grade 3 had a significant increase compared with normal cartilage and early OA cartilage (grade 1 and 2).

Therefore, it might be speculated that the reflection coefficient of the cartilage surface could be a more sensitive index to distinguish the early OA grades than the surface roughness index and the reflection coefficient of the cartilage-bone interface. The integrated analysis of these three parameters in diagnosis of cartilage degeneration not only evaluates the surface morphology (surface roughness), but also assesses the impact of the composition changes by measurement of the reflection coefficients of both the cartilage surface and the cartilage-bone interface. Thus, more accurate diagnostic results may be obtained.

Two limitations of this study require further investigations. First, the OA grades were only evaluated subjectively by three pathologists in this study. The quantitative analysis of changes in compositions and structural parameters such as PG content and cartilage thickness and the sequent study in their relationships to ultrasound parameters are needed. Secondly, the numbers of the samples with different OA grades were uneven, especially the number of OA cartilage with grade 3 was small (N = 6). The small number of samples may have some impacts on the statistical results. Thus further studies with large number of samples are needed.

## Conclusion

The UBM measurements reflect the changes in the surface roughness index and the ultrasound reflection coefficients of the cartilage samples with different OA grades. This study suggests that these two ultrasound acoustic parameters have potential to become objective criteria in OA grading.

## Competing interests

All authors have no competing interests according to the products used.

## Authors' contributions

HJN, YXW, DYL, and YBF were involved in the design of the study and performed the statistical analysis. HJN, QW, and WFC were responsible for drafting the paper and revising it. And all authors commented on the draft. All authors have read and approved the final manuscript.

## Pre-publication history

The pre-publication history for this paper can be accessed here:

http://www.biomedcentral.com/1471-2474/13/34/prepub
